# *In-vitro* Neurotoxicity of Two Malaysian Krait Species (*Bungarus candidus* and *Bungarus fasciatus*) Venoms: Neutralization by Monovalent and Polyvalent Antivenoms from Thailand

**DOI:** 10.3390/toxins6031036

**Published:** 2014-03-12

**Authors:** Muhamad Rusdi Ahmad Rusmili, Tee Ting Yee, Mohd Rais Mustafa, Iekhsan Othman, Wayne C. Hodgson

**Affiliations:** 1 Monash Venom Group, Department of Pharmacology, Faculty of Medicine, Nursing and Health Sciences, Clayton, Victoria 3168, Australia; E-Mail: muhamad.rusmili@monash.edu; 2 Jeffrey Cheah School of Medicine and Health Sciences, Monash University Sunway Campus, Bandar Sunway 46150, Malaysia; E-Mails: ting.tee@monash.edu (T.T.Y.); iekhsan.othman@monash.edu (I.O.); 3 Department of Basic Medical Sciences, Kulliyyah of Pharmacy, International Islamic University Malaysia, Bandar Indera Mahkota 23800, Malaysia; 4 Department of Pharmacology, Faculty of Medicine, University of Malaya, Kuala Lumpur 59100, Malaysia; E-Mail: rais@um.edu.my

**Keywords:** venom, krait, snake, neurotoxicity, antivenom, *Bungarus fasciatus*, *Bungarus candidus*

## Abstract

*Bungarus candidus* and *Bungarus fasciatus* are two species of krait found in Southeast Asia. Envenoming by these snakes is often characterized by neurotoxicity and, without treatment, causes considerable morbidity and mortality. In this study, the *in vitro* neurotoxicity of each species, and the effectiveness of two monovalent antivenoms and a polyvalent antivenom, against the neurotoxic effects of the venoms, were examined in a skeletal muscle preparation. Both venoms caused concentration-dependent inhibition of indirect twitches, and attenuated responses to exogenous nicotinic receptor agonists, in the chick biventer preparation, with *B. candidus* venom being more potent than *B. fasciatus* venom. SDS-PAGE and western blot analysis indicated different profiles between the venoms. Despite these differences, most proteins bands were recognized by all three antivenoms. Antivenom, added prior to the venoms, attenuated the neurotoxic effect of the venoms. Interestingly, the respective monovalent antivenoms did not neutralize the effects of the venom from the other Bungarus species indicating a relative absence of cross-neutralization. Addition of a high concentration of polyvalent antivenom, at the *t_90_* time point after addition of venom, partially reversed the neurotoxicity of *B. fasciatus* venom but not *B. candidus* venom. The monovalent antivenoms had no significant effect when added at the *t_90_* time point. This study showed that *B. candidus* and *B. fasciatus* venoms display marked *in vitro* neurotoxicity in the chick biventer preparation and administration of antivenoms at high dose is necessary to prevent or reverse neurotoxicity.

## 1. Introduction

Snakes from the *Bungarus* genus, commonly known as kraits, are nocturnal venomous snakes found in many parts of Asia. There are three species of *Bungarus* found in Malaysia: *Bungarus candidus* (Malayan krait), *Bungarus fasciatus* (banded krait) and *Bungarus flaviceps* (red-headed krait) [1]. There are two subspecies of *Bungarus flaviceps*, *Bungarus flaviceps flaviceps* and *Bungarus flaviceps baluensis* [2]. A high number of krait envenoming cases have been reported in India, Sri Lanka, Thailand and Vietnam [3,4,5,6,7,8]. However, envenoming by kraits is relatively uncommon in Malaysia [9,10].

Neurotoxicity characterizes systemic envenoming by kraits [11] and has been attributed to the presence of two major classes of neurotoxins in the venom, *i.e.*, presynaptic and postsynaptic neurotoxins [7,12,13]. Presynaptic neurotoxins disrupt the release of the neurotransmitter acetylcholine from the nerve terminal while postsynaptic neurotoxins inhibit the binding of acetylcholine to the nicotinic receptors on the skeletal muscle [12]. Krait venoms have also been shown to contain acetylcholinesterase, hyalurodinase and L-amino acid oxidase, which have been hypothesized to enhance toxin penetration and contribute to the neurotoxic effect of the venoms [14]. 

Untreated respiratory muscle paralysis is the main cause of lethality in krait envenoming [3,15]. Morbidities in the form of permanent brain damage due to anoxia, cerebral ataxia, paraplesia, mydriasis, problems effecting micturation and causing constipation have also been reported in victims of krait bites [4,16]. Treatment options for systemic krait envenoming include administration of antivenom and supportive respiratory assistance. High doses of antivenom are often used in krait envenomings with limited success in reversing the neurotoxic symptoms [4,17]. The Queen Saovabha Memorial Institute (The Thai Red Cross Society; Bangkok, Thailand) produces *B. fasciatus* antivenom (BFAV) and *B. candidus* antivenom (BCAV) [18]. In addition, the Institute produces Neuro Polyvalent Snake antivenom (NPAV) for elapid envenoming which covers not only *B. candidus* and *B. fasciatus* but also *Ophiophagus hannah* and *Naja kouthia* [19].

The efficacy of NPAV against Malaysian elapid species and African cobras has been shown previously in *in vivo* experiments [19]. BFAV has been shown to have intraspecific neutralizing effects when tested against three species of krait found in Thailand [20]. However, BFAV was not effective when administered in patients envenomed by *B. candidus* [4]. BCAV reduced hospitalization time in patients envenomed by *B. candidus* in Thailand but there has been no study on the cross-neutralizing activity of this antivenom against other krait venoms [18]. The inability of polyvalent antivenoms, containing antibodies raised against the venoms of different *Bungarus* species, to neutralize neurotoxicity has been previously documented [5,21]. Geographical variations in venom composition have also been found to be a key factor in determining antivenom efficacy [22,23,24]. Pharmacological, biochemical and proteomic studies on various snake species showed that there are differences in the activities and composition of venom from the same snake species from different localities [23,25,26,27]

In the current study, the *in vitro* neurotoxic activity of Malaysian *B. candidus* and *B. fasciatus* venoms was assessed in the indirectly-stimulated chick biventer cervicis nerve-muscle preparation. The time to 90% inhibition of the original twitch height (*i.e.*, *t_90_*) was used to quantify the neurotoxicity of the venoms [28]. The neutralizing capability of two monovalent antivenoms, BCAV and BFAV, and a polyvalent antivenom, NPAV, against Malaysian *B. candidus* and *B. fasciatus* was also assessed. In addition, the cross-neutralising capability of the monovalent antivenoms was evaluated.

## 2. Results

### 2.1. Chick Biventer Cercivis Nerve-Muscle Preparation

**Figure 1:**
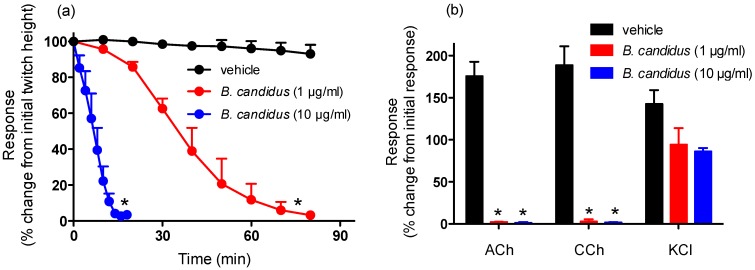
Effect of *B. candidus* venom on (**a**) indirect twitches of the chick biventer cervicis nerve-muscle preparation and (**b**) responses to exogenous agonists. *: *P* < 0.05, significantly different from vehicle (*n* = 3–4, one-way ANOVA for (**a**) and two-way ANOVA for (**b**)).

**Figure 2:**
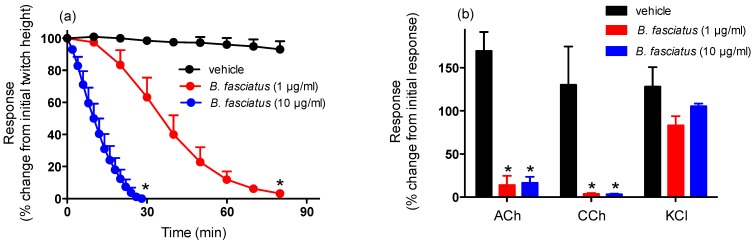
Effect of *B. fasciatus* venom on (**a**) indirect twitches of the chick biventer cervicis nerve-muscle preparation and (**b**) responses to exogenous agonists. *: *P* < 0.05, significantly different from vehicle (*n* = 3–4, one-way ANOVA for (**a**) and two-way ANOVA for (**b**)).

*B. candidus* (1 and 10 µg/mL; Figure 1a) and *B. fasciatus* (1 and 10 µg/mL; Figure 2a) venoms abolished indirect twitches of the chick biventer cervicis nerve-muscle preparation. The time required for the twitches to be reduced by 90% (*i.e.*, *t*_90_) were: *B. candidus* venom; 1 µg/mL, 61 ± 8 min; 10 µg/mL, 13 ± 4 min and for *B. fasciatus* venom; 1 µg/mL, 62 ± 5 min; 10 µg/mL, 22 ± 5 min. Repetitive washing with physiological salt solution, commencing at the *t_90_* time point after the addition of the venoms (1 µg/mL), did not reverse the neurotoxicity induced by either venom (data not shown).

*B. candidus* (1 and 10 µg/mL; Figure 1b) and *B. fasciatus* (1 and 10 µg/mL; Figure 2b) venoms almost completely abolished responses to acetylcholine (ACh) and carbachol (CCh) but not responses to potassium chloride (KCl). 

### 2.2. Antivenom Studies

**Figure 3:**
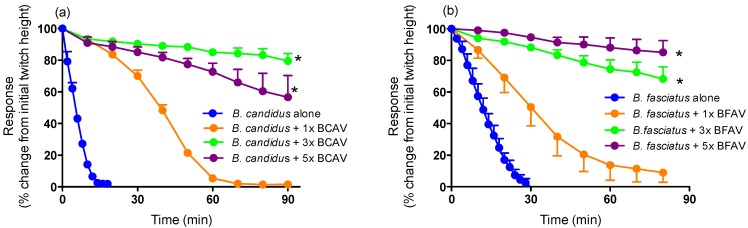
Effect of prior addition of increasing concentrations of (**a**) *B. candidus* antivenom (BCAV) on the neurotoxic effects of *B. candidus* venom (10 µg/mL) or (**b**) *B. fasciatus* antivenom (BFAV) on the neurotoxic effects of *B. fasciatus* venom (10 µg/mL) in the chick biventer cervicis nerve-muscle preparation. *: *P* < 0.05, significantly different than the respective venom alone (*n* = 3–4, one-way ANOVA).

Pre-incubation of monovalent *B. candidus* and *B. fasciatus* antivenoms at 1×, 3× or 5× the recommended titres (*i.e.*, 1 mL per 0.6 mg of *B. fasciatus* venom and 1 mL per 0.4 mg of *B. candidus* venom) prior to the addition of *B. candidus* (10 µg/mL) or *B. fasciatus* (10 µg/mL) venoms, caused either a marked delay in the time to abolish twitches (*i.e.*, 1× each antivenom) or prevented twitch inhibition (*i.e.*, 3× and 5× concentrations of each antivenom) (Figure 3a,b).

**Figure 4:**
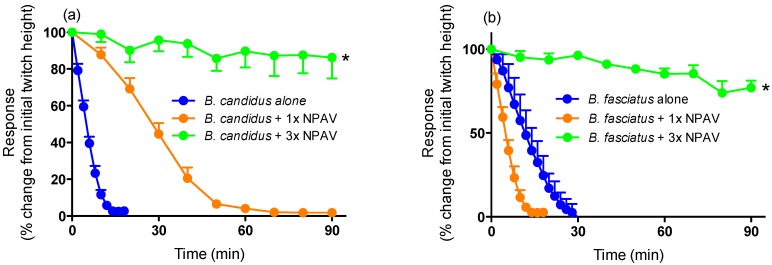
Effect of prior addition of increasing concentrations of Neuro Polyvalent antivenom (NPAV) on the neurotoxic effects of (**a**) *B. candidus* venom (10 µg/mL) or (**b**) *B. fasciatus* venom (10 µg/mL) in the chick biventer cervicis nerve-muscle preparation. *: *P* < 0.05, significantly different than the respective venom alone (*n* = 3–4, one-way ANOVA).

Pre-incubation with 3× the recommended titre of NPAV also markedly attenuated the reduction in twitch height induced by *B. candidus* (10 µg/mL) and *B. fasciatus* (10 µg/mL) venoms (Figure 4a,b).

**Figure 5:**
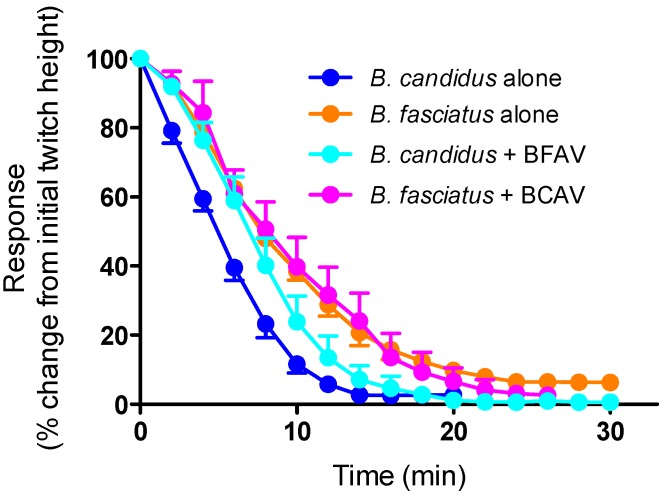
Effect of prior addition of *B. candidus* antivenom (3×; BCAV) or *B. fasciatus* antivenom (3×; BFAV) on the neurotoxic effects of *B. fasciatus* (10 µg/mL; *n* = 3) or *B. candidus* (10 µg/mL; *n* = 3) venoms, respectively, in the chick biventer cervicis nerve-muscle preparation.

The prior addition of monovalent BFAV (3× titre) was unable to prevent the reduction in twitches induced by the subsequent addition of *B. candidus* venom (10 µg/mL) (Figure 5). Similarly, the prior addition of monovalent BCAV (3× titre) was unable to prevent the reduction in twitches induced by the subsequent addition of *B. fasciatus* venom (10 µg/mL) (Figure 5).

**Figure 6:**
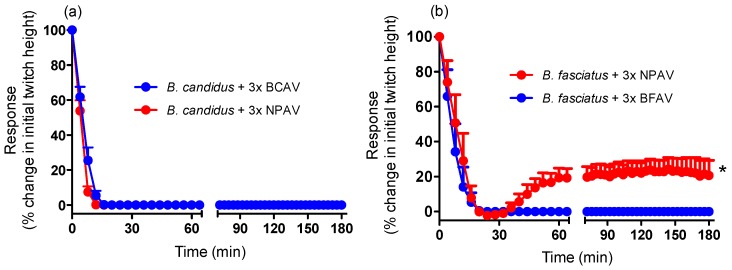
Effect of (**a**) *B. candidus* antivenom (3×; BCAV) or Neuro Polyvalent antivenom (3×; NPAV), added at the *t_90_* time point, on the neurotoxic effects of *B. candidus* (10 µg/mL) venom or (**b**) *B. fasciatus* antivenom (3×; BFAV) or Neuro Polyvalent antivenom (3×; NPAV), added at the *t_90_* time point, on the neurotoxic effects of *B. fasciatus* (10 µg/mL) venom in the chick biventer cervicis nerve-muscle preparation. *: *P* < 0.05, significantly different from zero, one sample t-test (*n* = 3).

Neither monovalent antivenom (3× recommended titre) significantly restored twitch height when added at the *t_90_* time point. However, NPAV produced a small but significant increase in twitch height for *B. fasciatus* venom when added at the *t_90_* time point *i.e.*, approximately 20% recovery of twitch height (Figure 6b).

### 2.3. SDS-PAGE and Western Blot

**Figure 7:**
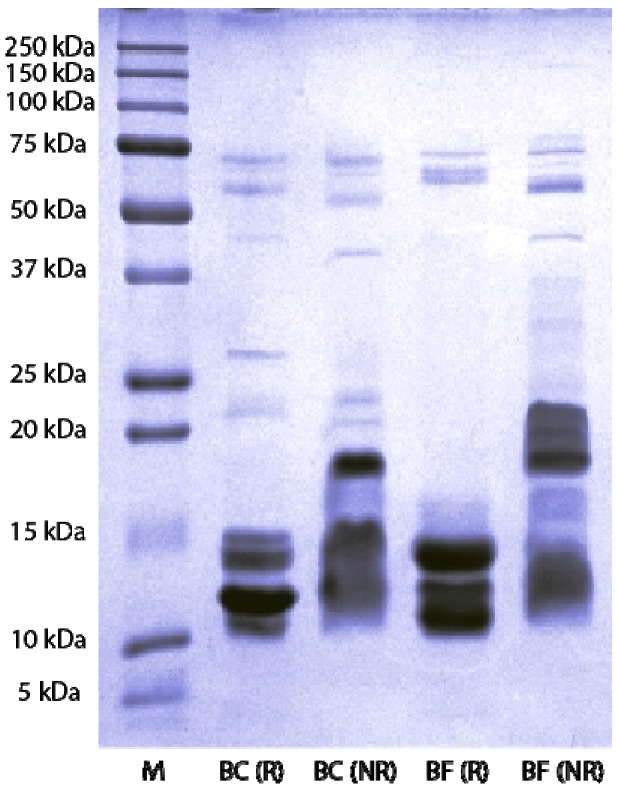
SDS-PAGE of venoms on a 12% gel. Venoms were treated in reducing and non-reducing buffer prior to loading and stained with Coomassie blue. M indicates the protein marker lane, BC(R) indicates *B. candidus* venom treated with reducing buffer and BC(NR) indicates *B. candidus* venom treated with non reducing sample buffer. BF(R) indicates *B. fasciatus* venom treated with reducing buffer and BF(NR) indicates *B. fasciatus* venom treated with non-reducing sample buffer.

SDS-PAGE analysis showed that *B. candidus* and *B. fasciatus* venoms consist of proteins of MW ranging from 10 to 75 kDa (Figure 7). Non-reduced venoms possessed a greater number of protein bands compared to reduced venoms. Thick and high intensity bands clumped together were observed in the MW range of 10–15 kDa in non-reduced *B. candidus* venom and 15–20 kDa in non-reduced *B. fasciatus* venom. No protein band was observed within the range of 20–50 kDa in reduced *B. fasciatus* venom. A single band was observed within the 150–250 kDa range in non-reduced *B. fasciatus* venom.

**Figure 8:**
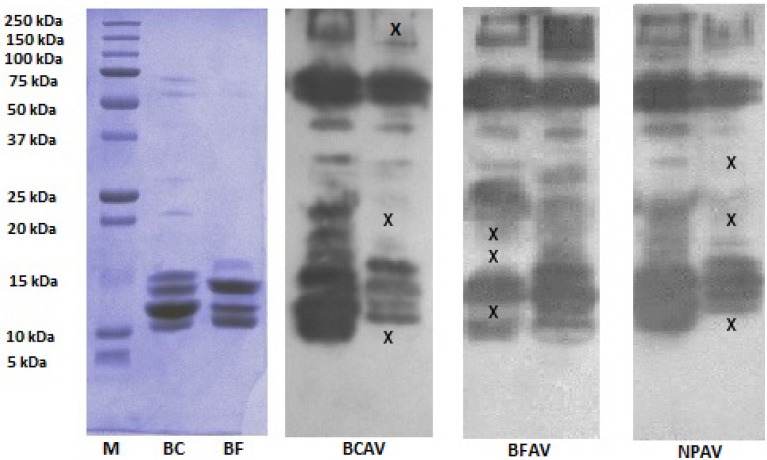
SDS-PAGE and western blot of venoms. M indicates the protein marker lane, BC (*B. candidus*) and BF (*B. fasciatus*) indicate reduced venoms in 12% gel. BCAV: reduced venoms incubated with monovalent *B. candidus* antivenom; BFAV: reduced venoms incubated with *B. fasciatus* antivenom; NPAV: reduced venoms incubated with neuro polyvalent antivenom. X indicates protein bands that were not detected based in the SDS-PAGE and in the membrane that was treated with the venom’s monovalent antivenom.

Western blotting of the gels with BCAV, BFAV or NPAV showed differences in the immunoreactivity profile against some protein bands in the venoms (Figure 8). Proteins belonging to the 15–50 kDa and 100–150 kDa ranges, previously not detected during SDS-PAGE, were identified by western blotting. The monovalent antivenoms were able to react with most of the protein bands from their respective species within the 15–20 kDa and 100–250 kDa weight ranges. 

## 3. Discussion

This study showed that both *B. candidus* and *B. fasciatus* venoms cause marked *in vitro* neurotoxicity. Based on t_90_ values, *B. candidus* was more potent than *B. fasciatus* venom. These studies are comparable to the results of previous lethality studies [14], although we caution against comparing the results of *in vitro* experiments to *in vivo* experiments. Contractile responses to ACh and CCh, but not KCl, were significantly attenuated by both venoms, indicating the likely presence of postsynaptic neurotoxins and an apparent lack of myotoxic activity. Repetitive washing with physiological salt solution did not reverse the neurotoxic effects of either venom, suggesting that the toxins inducing this effect were irreversible or pseudo-irreversible. It also supports the previous findings on the detection and isolation of potent presynaptic and postsynaptic neurotoxins in *B. candidus* and *B. fasciatus* venoms [7,15,29]. Pseudo-irreversible antagonism occurs when the agonist (e.g., acetylcholine released from the nerve terminal) and antagonist (e.g., post-synaptic neurotoxins in the venom) compete for the receptor (e.g., skeletal muscle nicotinic receptor) but the antagonist dissociates from the receptor so slowly (*i.e.*, slow off-rate antagonist) that the agonist cannot achieve equilibrium with the receptor.

SDS-PAGE analysis showed that both venoms consist of a complex mixture of proteins with different molecular weights. The lower number of protein bands in reduced venoms compared to non-reduced venoms indicates the presence of proteins complexes in the venoms. Reduced presynaptic phospholipase and three-finger toxins in krait venoms are likely to be responsible for the higher number of bands within the 10–15 kDa range when the venoms are reduced [13,30].

Western blotting against BCAV, BFAV and NPAV showed that there are differences in the immunoreactivity of protein components in the venoms. The absence of several bands in the blots indicate that not all proteins in the venoms react with antivenoms and these components may contribute to the lack of effectiveness of the antivenoms. However, a single band in SDS-PAGE may contain several proteins even after fractionation [31]. Therefore, there is a possibility that immunoreactive proteins may be masked with proteins that do not react with the antivenom within the same band. In addition, caution should be displayed when extrapolating the data from SDS-PAGE and western blot experiments as the bands correspond to denatured proteins and we cannot exclude the possibility that the antivenoms may recognize some proteins *in vivo* but not the western blot.

The efficacy of the antivenoms used in the current study have not previously been examined *in vitro*. Interestingly, a relatively high concentration (*i.e.*, at least three times the recommended titre) of all antivenoms was required to prevent attenuation of indirect twitches. This may indicate that the composition of the Malaysian krait venoms are slightly different than the venoms used in the production of the antivenoms. Indeed, differences in the composition/activity of *B. candidus* and *B. fasciatus* venoms from different populations have been previously reported [7,32]. It is also possible that higher concentrations of antivenoms are required due to the lower immunogenicity of low molecular neurotoxins [22,33] which are responsible for the *in vitro* effects. The antivenoms were also largely ineffective, even at high concentrations, when added at the *t_90_* time point. The inability of the antivenoms to markedly reverse the neurotoxic effects, after venom administration, is likely to be related to the irreversible effects of presynaptic neurotoxins in the venoms [34]. 

The lack of effect of the monovalent antivenoms against the venom of the other krait species (*i.e.*, BCAV against *B. fasciatus* venom, and vice versa) indicates a relative absence of cross-neutralization. This finding supports clinical observations where no beneficial outcome was seen when *B. fasciatus* antivenom was administered to patients envenomed by *B. candidus* [4] despite observed neutralization in animals [20]. The absence of cross-neutralization by either monovalent antivenom indicates that interspecies variation exists in the composition of the neurotoxins in the venoms. Therefore, it is possible that the immunoreactivity of some proteins observed in the western blot was due to binding of the antivenoms to invariant amino acid positions in the toxins [35] or at a position that had been modified during sample pretreatment [36]. Indeed, phospholipase A_2_ neurotoxins and three finger toxin families are known to have similar invariant structural arrangements within the families [37,38]. 

## 4. Experimental Section

### 4.1. Venoms and Antivenoms

*B. candidus* and *B. fasciatus* venoms were a gift from Mr. Zainuddin Ismail (Perlis, Northwest Peninsular Malaysia). The snakes were collected in states of Kedah and Perlis, Northwest Peninsular Malaysia. The venoms from 10 snakes from each species were obtained and pooled by allowing snakes to bite plastic containers covered with Parafilm. After collection, the pooled venoms were transported on ice back to the laboratory, frozen at −80 °C and then freeze-dried. Freeze-dried venom samples were weighed, labeled and stored at −20 °C prior use. When required, the venoms were weighed and dissolved in Milli-Q water. *B. candidus* antivenom (Lot No.:BC00112) and *B. fasciatus* antivenom (Lot No.:BKC0108), and Neuro Polyvalent antivenom (Lot No.:KP00109) were purchased from Queen Saovabha Red Cross Society, Bangkok, Thailand. The freeze-dried antivenoms were dissolved with pharmaceutical grade water supplied by the manufacturer. The dissolved antivenoms were stored at 4 °C prior to use.

### 4.2. Chemicals and Drugs

The following were purchased from Sigma Aldrich (St. Louis, MO, USA): acetylcholine (ACh), carbamylcholine chloride (CCh), *d*-tubocurarine, bovine serum albumin.

### 4.3. Protein Quantification by Bicinchoninic Acid Assay (BCA)

Protein quantification was performed as instructed by the manufacturer (Pierce Biotechnology, Rockford, IL, USA). Approximately 25 µL of sample or standard was loaded onto a 96-well plate in triplicate, then 200 µL reagent buffer mix was added to each well. The plate was incubated at 37 °C for 30 min, then read at 562 nm using a plate reader spectrophotometer (VersaMax Elisa Microplate reader, Molecular Device, Sunnyvale, CA, USA). The protein concentration was determined from the standard curve.

### 4.4. Indirectly-Stimulated Chick Biventer Cervicis Nerve-Muscle Preparation (CBCM)

The experiments using this preparation were conducted using a method previously described [21,23,35]. Male chicks (5–12 days old) were killed by CO_2_ inhalation before the biventer nerve-muscle preparations were dissected and mounted in 5 mL organ baths aerated with carbogen (5% CO_2_ and 95% O_2_). The organ baths were filled with physiological salt solution of the following composition: NaCl, 118.4 mM; KCl, 4.7 mM; MgSO_4_, 1.2 mM KH_2_PO_4_, 1.2 mM; CaCl_2_, 2.5 mM; NaHCO_3_, 25 mM and glucose, 11.1 mM. The tissues were stimulated every 10 s with pulses of 0.2 ms duration at a supramaximal voltage (10–20 V) using a Grass S88 stimulator attached to silver ring electrodes. Indirect stimulation of the tissues was confirmed by the abolishment of twitches by *d*-tubocurarine (10 µM), which was then washed out until twitches were restored. In the absence of electrical stimulation, responses to acetylcholine (ACh; 1 mM for 30 s), carbachol (CCh; 20 µM for 60 s) and KCl (40 mM for 30 s) were obtained. Responses were recorded on a PowerLab (ADInstruments, Bella Vista, NSW, Australia).

For antivenom studies, antivenom was added into the organ bath 10 min prior to the addition of venom or after the addition of venom at the t_90_ time point.

### 4.5. Sodium Dodecyl Sulphate Polyacylamide Gel Electrophoresis (SDS-PAGE)

Sodium dodecyl sulphate polyacrylamide gel electrophoresis (SDS-PAGE) was conducted in 12% polyacrylamide gel by using the method previously described [39]. The gels were then stained with Coomassie blue and de-stained by using 10% acetic acid in water. Kaleidoscope Protein Marker (Bio-Rad, Hercules, CA, USA) was used in the gel for molecular weight protein marker. The gel was scanned using the ChemiDoc XRS Imaging System (BioRad, Hercules, CA, USA).

### 4.6. Western-Blot

Venoms (10 µg) were loaded and electrophoresed in 12% separating SDS-PAGE gel with 5% stacking gel as described in section 4.5. The venom was transferred onto polyvinylidene difluoride (PVDF) membrane (Merck Milipore, Billerica, MA, USA) by semi-dry electroblotting (BioRad, Hercules, CA, USA) at 10 V for 45 min. The membrane was then blocked in Tris-buffered saline with 1% Tween 20 (TBST) buffer (20 mM Tris, 0.5 M NaCl and 0.5% Tween-20) supplemented with 5% skim milk for 1 h at room temperature. Primary antibodies (*i.e.*, Thai Red Cross Society’s *B. fascistus* antivenom, *B. candidus* antivenom or Neuro Polyvalent antivenom diluted 1:500 fold in TBST with 5% skim milk) were then added and the membrane incubated overnight at 4 °C. The membrane was then washed three times for 15 min with TBST buffer. Secondary antibody (*i.e.*, goat anti-horse IgG secondary antibody conjugated with horseradish peroxidase (Santa Cruz Biotechnology Inc., Dallas, TX, USA) diluted in 1:5000 with TBST buffer with 5% skim milk) was added to the membrane and left for 45 min at room temperature. The membrane was then washed three times for 10 min and the membrane incubated with Immobilon western chemiluminiscent (ECL) substrate (Merck Miliprore, Billerica, MA, USA) as described by the manufacturer. After incubation, the excess ECL reagent was removed and the membrane was exposed to x-ray film (Fujifim, Melbourne, Australia) to visualize the bands. The film and the membrane were scanned using the ChemiDoc XRS Imaging System (BioRad, Hercules, CA, USA).

### 4.7. Data Analysis

Changes in twitch height are expressed as a percentage of the original twitch height (*i.e.*, prior to the addition of venom). Changes in the magnitude of contractile responses to exogenous agonists are expressed as a percentage of the original response to the agonist prior to the addition of venom. Data were analyzed by using a one-way analysis of variance (ANOVA) or two-way ANOVA followed by Dunnet's multiple comparison test (GraphPad Prism 6, La Jolla, CA, USA).

## 5. Conclusions

In conclusion, we found that Malaysian *Bungarus candidus* and *Bungarus fasciatus* venoms display marked *in vitro* neurotoxicity with *B. candidus* being slightly more potent than *B. fasciatus*. The neurotoxic effect of these venoms was attenuated by their respective monovalent antivenoms, albeit at higher concentrations than recommended, but there was limited cross neutralization. The apparent presence of potent, but low immunogenic neurotoxins, requires more investigation to identify methods to enhance the efficacy of the antivenoms. The data suggests that the non-specific Neuro Polyvalent antivenom may be useful for the treatment of *B. candidus* or *B. fasciatus* envenoming if the biting species cannot be determined or the specific monovalent antivenom is unavailable. Given the irreversible nature of the neurotoxic effects of the venoms, it would appear that early administration of the appropriate antivenom would be advantageous. A clinical trial is needed to assess the appropriate dose of antivenom to be use in the treatment for envenoming by these Malaysian species. This study also showed that there is a need for further work to explore geographical variation of the venoms from these species. 
